# STK25 enhances hepatocellular carcinoma progression through the STRN/AMPK/ACC1 pathway

**DOI:** 10.1186/s12935-021-02421-w

**Published:** 2022-01-05

**Authors:** Yichao Zhang, Junhui Xu, Zhendong Qiu, Yongjun Guan, XiaoYi Zhang, Xin Zhang, Dongqi Chai, Chen Chen, Qinyong Hu, Weixing Wang

**Affiliations:** 1 Department of General Surgery, Renmin Hospital of Wuhan University, Wuhan, Hubei China; 2 Cancer Center, Renmin Hospital of Wuhan University, Wuhan, China; 3 Intensive Care Unit, ZhongNan Hospital of Wuhan University, Wuhan, China

**Keywords:** STK25, STRN, HCC, Lipid metabolism, Cancer, Therapy

## Abstract

**Background:**

Serine/threonine protein kinase 25 (STK25) plays an important role in regulating glucose and insulin homeostasis and in ectopic lipid accumulation. It directly affects the progression and prognosis of nonalcoholic fatty liver disease (NAFLD). However, the effects of STK25 on lipid metabolism in hepatocellular carcinoma (HCC) remain unexplored. The aim of this study was to investigate the role of STK25 in HCC and to elucidate the underlying mechanisms.

**Methods:**

Immunohistochemistry was used to measure the expression of STK25 in hepatic tissues of HCC patients, and public datasets were used as supplementary material for predicting the expression of STK25 and the prognosis of patients with HCC. The interaction between STK25 and striatin (STRN) was determined by the STRING database, immunohistochemistry and western blot analyses. The involved signaling pathway was detected by the KEGG database and western blot. Moreover, the biological behaviors of the HCC cells were detected by wound healing assays, Transwell invasion assays and oil red O staining. Finally, it was verified again by xenograft model.

**Results:**

STK25 is highly expressed in HCC patients and is associated with poor prognosis. STK25 knockdown inhibited the HCC cell invasion and proliferation, promotes apoptosis. Consistently, STK25 knockdown inhibited tumor growth in xenograft mouse model. Besides, STK25 deficiency decreased lipid synthesis, energy reserve, epithelial-mesenchymal transition (EMT) by down-regulating lipid metabolism signaling pathway. STRN could reverse the change of lipid metabolism.

**Conclusions:**

Our results demonstrated that STK25 interacted with STRN to regulates the energy reserve and EMT via lipid metabolism reprogramming. Accordingly, high expression of STK25 may be associated with HCC patients and poor prognosis, which implicates STK25 could be a potential target for lipid metabolism in cancer therapy.

**Supplementary Information:**

The online version contains supplementary material available at 10.1186/s12935-021-02421-w.

## Background

Liver cancer is the sixth most common cancer in the world and has a high mortality rate, ranking second in the world following lung cancer. According to statistics, the mortality rate of liver cancer increased by 4.6% from 2005 to 2015 [1]. Hepatocellular carcinoma (HCC) accounts for more than 80% of primary liver cancers. Most of them develop from chronic persistent hepatitis caused by viruses. In China, 93% of HCC cases are caused by HBV infection [2]. With the development of society, people's lifestyle and nutritional status have been greatly improved. Nonalcoholic fatty liver disease (NAFLD) has become the most common liver disease in most developed countries and has become the main risk factor for HCC [3, 4]. However, the mechanisms of how NAFLD develops into HCC remain unclear. Researchers have pointed out that factors that are closely related to NAFLD, such as obesity, diabetes, and iron deposition, are independent risk factors for HCC. Therefore, it is of great clinical significance to explore how the liver transforms from benign steatosis to liver cancer and achieve early interventions.

Serine/threonine protein kinase 25 (STK25), also known as YSK1 and SOK1, is a member of the germinal center kinase subfamily III (GCKIII), and other members of the same family are MST3 (mammalian Ste20-like kinase 3) and MST4 [5]. In studies on NAFLD, STK25 plays an important role in regulating glucose and insulin homeostasis and ectopic lipid accumulation, and it directly affects the progression and prognosis of NAFLD [6]. According to the human cancer databases, focal deletion of STK25 is also very common in human cancers, such as cervical squamous cell carcinoma, bladder urothelial carcinoma, and head and neck squamous cell carcinoma, and it can be regulated by different mechanisms [7]. Su et al. proposed in 2018 that STK25 negatively regulated tumor cell proliferation by downregulating the Golph3-dependent mTOR pathway and inhibiting glycolysis, which linked tumor metabolism with STK25 for the first time [8]. In other words, STK25 is closely related to tumor and energy metabolism.

Striatin (STRN) was first discovered in the synapses of rats in 1996; STRN is concentrated in the striatum and motor neurons and is highly expressed in the central and peripheral nervous systems. Its family members include STRN, SG2NA (STRN3) and Zinedin (STRN4) [9]. STRN is widely expressed in the lungs, liver, kidney, bones, etc., and participates in the regulation of various physiological processes [10]. Protein phosphatase 2A (PP2A) is a multifunctional serine/threonine phosphatase that is composed of skeleton subunits (PP2A A, 65 kD), regulatory subunits (PP2A B, 50–130 kD) and catalytic subunits (PP2A C, 36 kD), so it is also called heterotrimeric protein phosphatase. The striatin family is one of the B subunit members of PP2A. Without the activation of other B subunit members, the striatin family binds the PP2A A/C dimer to change its activity to form the PP2A holoenzyme (Fig. 5a), which is very important for the dephosphorylation and inactivation of GCKIII [11].

A large number of studies have emphasized the role of PP2A as a tumor suppressor and it plays an important role in carcinogenesis, suggesting that the destruction of the PP2A holoenzyme may contribute to the development of cancer. The mechanisms include somatic mutations, loss of heterozygosity and/or decreased expression of the PP2A subunits, increased expression of endogenous PP2A inhibitors and C subunit phosphorylation/methylation changes [12]. Moreover, it was found that adropin can activate the AMPK via suppression of PP2A and inhibits the liver glucose production in insulin-resistant hepatocytes [13]. Therefore, how STRN participates in the interaction with PP2A subunits and thus affects the liver lipid metabolism pathways is worthy of further study.

In this study, we analysed the expression of STK25 in hepatic tissues of HCC patients, and predict the correlation between STK25 and prognosis as a supplement. After clarifying the importance of STK25 in HCC patients, we investigated the potential molecular mechanism by establishing cell lines and xenograft mouse model. Then, we preliminarily confirmed that STRN could reverse the effect of STK25 depletion through AMPK/ACC1 pathway, which suggested that STK25 enhances hepatocellular carcinoma progression through the STRN/AMPK/ACC1 pathway.

## Materials and methods

### Human samples

Patients hospitalized in the Department of General Surgery at Renmin Hospital of Wuhan University from January 2019 to October 2020 were included in this study. The inclusion criteria were as follows: (1) Diagnosed by histopathological examination. (2) Not received radiotherapy, chemotherapy or other targeted therapy before surgery. The exclusion criteria were as follows: (1) Lipid metabolic abnormalities, such as severe hyperlipidemia, lipid storage disease and obesity. (2) Other systemic tumors. The use of the patient specimens was approved by the Ethics Committee of Wuhan University. Informed consent was obtained from the included patients.

### Data mining and collection

The data of liver cancer patients and RNA-seq expression results were downloaded from GEPIA (http://gepia.cancer-pku.cn/), HCCDB (http://lifeome.net/database/hccdb) [14] and TCGA. The cutoff values were identified by X-tile. The cell line expression matrix of liver tumors was obtained from the CCLE dataset (https://portals.broadinstitute.org/ccle/about) [15]. The above analysis was constructed by the R v4.0.3 software package ggplot2 (v3.3.3). The gene microarray with survival data (GSE14520) was downloaded from the GEO database (https://www.ncbi.nlm.nih.gov/geo/). Differentially expressed genes (DEGs) were screened by R software, and the pathway enrichment analysis of DEGs was performed with the DAVID, String, and Cytoscape software programs. Then, Gene Ontology (GO) enrichment analysis and Kyoto Encyclopedia of Genes and Genomes (KEGG) signaling pathway analysis were performed.

### Immunohistochemistry assay

For immunohistochemical staining of human livers, after deparaffinization, hydration, antigen retrieval, and serum blocking, hepatic sections were incubated overnight at 4 °C with mouse anti-YSK1 (1:100, Santa Cruz, sc-271196), anti-STRN (1:100, ABclonal, A7734), rabbit anti-E-cadherin (1:2000, proteintech, 20874-1-AP), anti-N-cadherin (1:2000, proteintech, 22018-1-AP) and anti-Ki67 (1:100, Bioss, bam-33070M). Goat anti-mouse/rabbit HRP secondary antibody (Maxim Biotech) was then added to the sections and incubated at room temperature for 1 h. The staining results were visualized using 3,5-diaminobenzidine.

### Immunofluorescence assay

For immunofluorescence staining of subcutaneous tumor, after deparaffinization, hydration, antigen retrieval, and serum blocking, tumor sections were incubated with primary antibodies diluted in PBS overnight at 4 °C. Alexa-conjugated secondary antibodies were used for fluorescent. Finally, slides were mounted via mounting medium with DAPI (Abcam, ab104139). The primary antibodies included ACC1 (1:100, Proteintech, 21923-1-AP), anti-ATP citrate lyase (1:100, Abcam, ab40793), Donkey anti-rabbit IgG H&L (Alexa Fluor 488) (1:200, Abcam, ab150073), Donkey anti-goat IgG H&L (Alexa Fluor 594) (1:200, Thermo Fisher Scientific, A-11058).

### Cell lines and cell culture

The HL-7702 (L02), HepG2, Huh7, and SMMC-7721 cell lines were purchased from the American Type Culture Collection (ATCC, Manassas, VA). The Dulbecco’s Modified Eagle’s Medium (DMEM, Servicebio, Wuhan, China) containing 10% fetal bovine serum (Gibco, Brazil), 100 U/mL penicillin, and 100 μg/mL streptomycin in a humidified atmosphere with 5% CO_2_ at 37 ℃.

### Plasmid transfections

To establish transient transfection of STK25 knockdown cell lines, overexpression cell line and STRN overexpression cell lines, cells were transfected with plasmids (Miaolingbio, China) or control shRNA. HCC cells were transiently transfected with the plasmids using Attractene Transfection Reagent (QIAGEN) according to the manufacturer’s instructions. For the table transfections, the cells were transfected with STK25 shRNA with GFP, the human STK25-targeting shRNA and control shRNA lentiviral plasmids were purchased from GeneChem (Shanghai, China). Stable knockdown cell lines were generated by lentivirus infection and selected with puromycin.

### Quantitative real-time PCR (qRT-PCR)

Total RNA of HCC tissue was extracted using the YPH EASY spin tissue/cell RNA quick extraction kit (YPH, Beijing China) following the manufacturer’s instructions. cDNA was prepared with a ReverTra Ace cDNA Reverse Transcription Kit (Toyobo, Osaka, Japan). Then, real-time PCR was carried out in a 15 µl reaction mixture containing diluted first strand cDNA and 1 × SYBR and primer mix (brand). The sequences of STK25 and GAPDH were as follows:

STK25: 5’-ATCAAGCAGTCGGCCTATGACT-3’ (Forward);

5’-CCTTGGCCAGCTCAATGG-3’(Reserve).

GAPDH: 5’-TGACTTCAACAGCGACACCCA-3’(Forward);

5’-CACCCTGTTGCTGTAGCCAAA-3’(Reserve).

### Western blot analysis

Liver and cell extracts were obtained using RIPA buffer (Beyotime, Guangzhou, China). After extraction, protein concentrations were determined by BCA. Equal amounts of proteins were separated via 10–12% SDS-PAGE and subsequently electrotransferred to PVDF membranes. After blocking with 5% nonfat milk, the blots were incubated with various primary antibodies, followed by HRP-conjugated secondary antibodies and then developed with ECL reagent (P0018FS, Beyotime, China). The primary antibodies included anti-YSK1 (1:500, Santa Cruz, sc-271196), anti-STRN (6) (1:500, Santa Cruz, sc-136084), anti-AMPK-α1 (1:1000, Beyotime, AF1627), anti-p-AMPK (1:1000, Abcam, ab133448), anti-ATP citrate lyase (1:2000, Abcam, ab40793), and anti-ACC1 (1:1000, Proteintech, 21923-1-AP), anti-beta actin (1:1000, Servicebio, GB11001), anti-E-cadherin (1:5000, proteintech, 20874-1-AP), anti-N-cadherin (1:5000, proteintech, 22018-1-AP), anti-snail (1:500, Proteintech, 13099-1-AP), anti-vimentin (1:2000, Proteintech, 10366-1-AP), HRP-conjugated goat anti-rabbit IgG (1:5000, Servicebio, GB23303), HRP-conjugated goat anti-mouce IgG (1:5000, Servicebio, GB23301). The densities of the proteins were quantified with ImageJ software.

### In vitro pull-down assay

For in vitro binding of STRN to STK25, total cell lysates of HepG2 or SMMC-7721 expressing STK25 were incubated with 20 μg of STRN, bound to glutathione-Sepharose beads for 1 h. The beads were then washed with lysis buffer three times and precipitated by the centrifugation. The precipitates were probed with anti-STK25 or anti-STRN antibody.

### Cell viability assay

After transfection, the cells were plated in 96-well plates at a density of 2 × 10^3^ cells per well and treated with complete medium (containing 10% FBS) at 37 ℃. To detect the cell viability, Cell Counting Kit-8 (Dojindo, CK04) was added to each well at 0, 24, 48, 72 and 96 h, respectively. Finally, the luminescence of each sample was determined at 450 nm using a microplate reader (PerkinElmer). The percentage of viable cells was estimated by comparison with the untreated controls. At least three independent experiments were performed.

### Detection of cell apoptosis by flow cytometry

Flow cytometry was conducted to evaluate apoptosis using PE Annexin V Apoptosis Detection Kit with 7-AAD (BD Biosciences) based on the manufacturer’s instructions. Changed the medium the day before, then collected all cells on the day of the test, incubated with Annexin V-PE and 7-AAD fluorescence dye for 30 min.

### Wound healing assay

We used a wound healing assay to determine cell migration ability. The transfected cells were cultured in 6-well plates, when the cells had just touched and fused, a wound was made in the monolayer of the cells with a 1 ml pipette tip. After the indicated treatment, the wounds were imaged at 0 h, 12 h and 24 h, and the difference was calculated via ImageJ software (version).

### Transwell invasion assay

Transwell migration assays were completed on 8-μm pore membranes in 24-well plates. A total of 5 × 10^5^ cells were seeded in DMEM high glucose medium with 10% FBS in the lower chamber as a chemoattractant. After 18 h, the cells that did not migrate were cleaned off the top of the membrane with a cotton swab, and the migrated cells were fixed, stained with crystal violet, and observed under a microscope. The average value of at least 6 visual fields was taken, and the experiment was repeated 3 times.

### Observation of lipid accumulation

Cells were fixed in 4% paraformaldehyde for 20 min and rinsed with 60% freezing isopropanol for 5 min. Then, the sections were stained for 30 min at room temperature with freshly prepared oil red O working solution (Solarbio, LOT:2018029), restained with hematoxylin (Servicebio, lot:183339) and then rinsed with flowing water. The sections were observed via phase-contrast microscopy (MicroPublisher 5.0 RTV, QIMAGING).

### Xenograft model

The animal studies were approved by the Ethics Committee of Renmin Hospital of Wuhan University. All the animal research procedures were performed according to the institutional ethical standards and/or those of the national research committee and according to the 1964 Helsinki declaration and its later amendments or comparable ethical standards.

Serum-free DMEM was used to wash the collected HepG2 cells, which were suspended in PBS. HepG2-shSTK25 and sh-Control cells (~ 5 × 10^6^) were injected subcutaneously in 5-weeks-old male BALB/c nude mice (Beijing Vital River Laboratory Animal Technology, China). Tumor growth was measured using calipers twice per week. In addition, 2–3 times per week, all the mice were weighed. At 4 weeks after tumor inoculation, the tumor weight was measured.

### Statistical analysis

Statistical analyses were performed using SPSS 17.0, and the data are shown as the mean ± s.d. Student’s t-test, one-way ANOVA followed by LSD post hoc test, the Kaplan–Meier method and chi-square tests were applied. P-value < 0.05 was regarded as significant. Fold-change (FC) and adjusted P-values were used to screen differentially expressed genes (DEGs). |log(FC)|≥ 1 and adj. P-val < 0.05 were defined as the screening criteria for the DEGs.

## Results

### General data analysis of the patients

A total of 29 patients with HCC in these two hospitals were included in this study. Among the 29 patients, 23 were male, and 6 were female, with an average age of 50.5 ± 8.9 years. In addition, we downloaded data from the TCGA, GEO and ICGC-LIRI-JP databases as supplementary material, including 371 tumor patients and 50 healthy patients (TCGA database), 64 tumor individuals and matched nontumor individuals (GEO database), 212 tumor tissue and 177 adjacent tissue (ICGC-LIRI-JP database). More database can be found in Table 1.

**Table 1:** STK25 expression from integrative molecular database of hepatocellular carcinoma

Source	P-value	Type	Numbers	Mean^#^
GSE22058	2.720e-18	HCC	100	9.864
		Adjacent	97	9.351
GSE25097	7.220e-29	HCC	268	0.6472
		Adjacent	243	0.4540
GSE36376	5.310e-56	HCC	240	8.260
		Adjacent	193	7.551
GSE14520	5.920e-28	HCC	225	4.749
		Adjacent	220	4.269
GSE10143	0.0001789	HCC	80	11.70
		Adjacent	82	11.26
GSE54236	0.004304	HCC	81	10.84
		Adjacent	80	10.58
GSE63898	2.210e-16	HCC	228	5.778
		Adjacent	168	5.413
GSE64041	0.000003230	HCC	60	8.510
		Adjacent	60	8.376
ICGC-LIRI-JP	1.520e-46	HCC	212	4.710
		Adjacent	177	3.935

Mean^#^: Relative expression of STK25 gene

The included databases, which has been uniformly processed, showed that the expression of STK25 gene in patients with HCC was higher than that in the control group. Statistically significant *p* values, *P* < 0.05

### Increased STK25 expression in HCC tissue

**Fig. 1:**
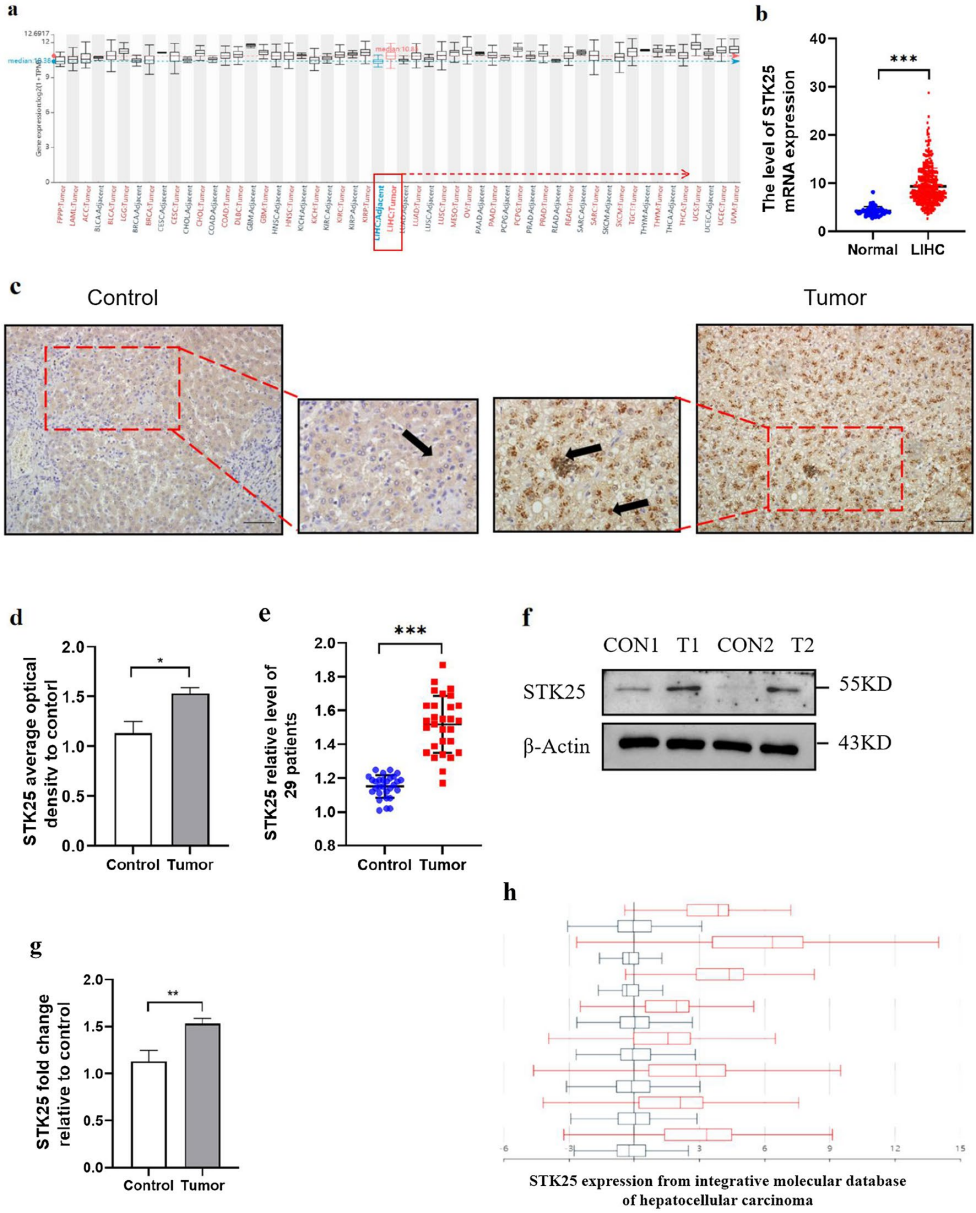
High expression of STK25 protein in HCC patients. (**a**) STK25 expression in different tissues (Adjacent vs Tumor). (**b**) STK25 expression in human HCC tissues from TCGA (Normal vs Tumor). (**c, d**) Representative staining (200 ×) and quantification of STK25 expression in liver of control and HCC patients, the thick black arrow indicated STK25. (**e**) STK25 expression of 29 patients in our center. (**f, g**) Representative western blot and quantification of STK25 in liver of control and HCC patients. (**h**) STK25 expression from integrative molecular database of hepatocellular carcinoma (As Table 1). CON, control. Data are expressed as mean ± SD. ^*^, *p* < 0.05. ^**^, *p* < 0.01. ^***^, *p* < 0.001. Scale bars, 50 μm

Using mRNA-seq data from GEPIA, we reviewed STK25 expression in different organs in TCGA, including adjacent and tumor. And compared with normal tissue, the average expression of STK25 in HCC was much higher (Fig. 1a, b). To confirm the difference in STK25 expression in the HCC tissues, we examined the tumor specimens and paracancerous tissues of 29 patients. The results of immunohistochemistry staining showed that hepatic tissues of HCC patients showed higher STK25 staining (Fig. 1c–e), which be consistent with the result of western blot (Fig. 1f, g). Furthermore, other databases in Table 1 also verified that STK25 was significantly overexpressed in HCC tissues (Fig. 1h).

### High STK25 expression is correlated with adverse clinicopathological characteristics and poor survival in HCC patients

**Fig. 2:**
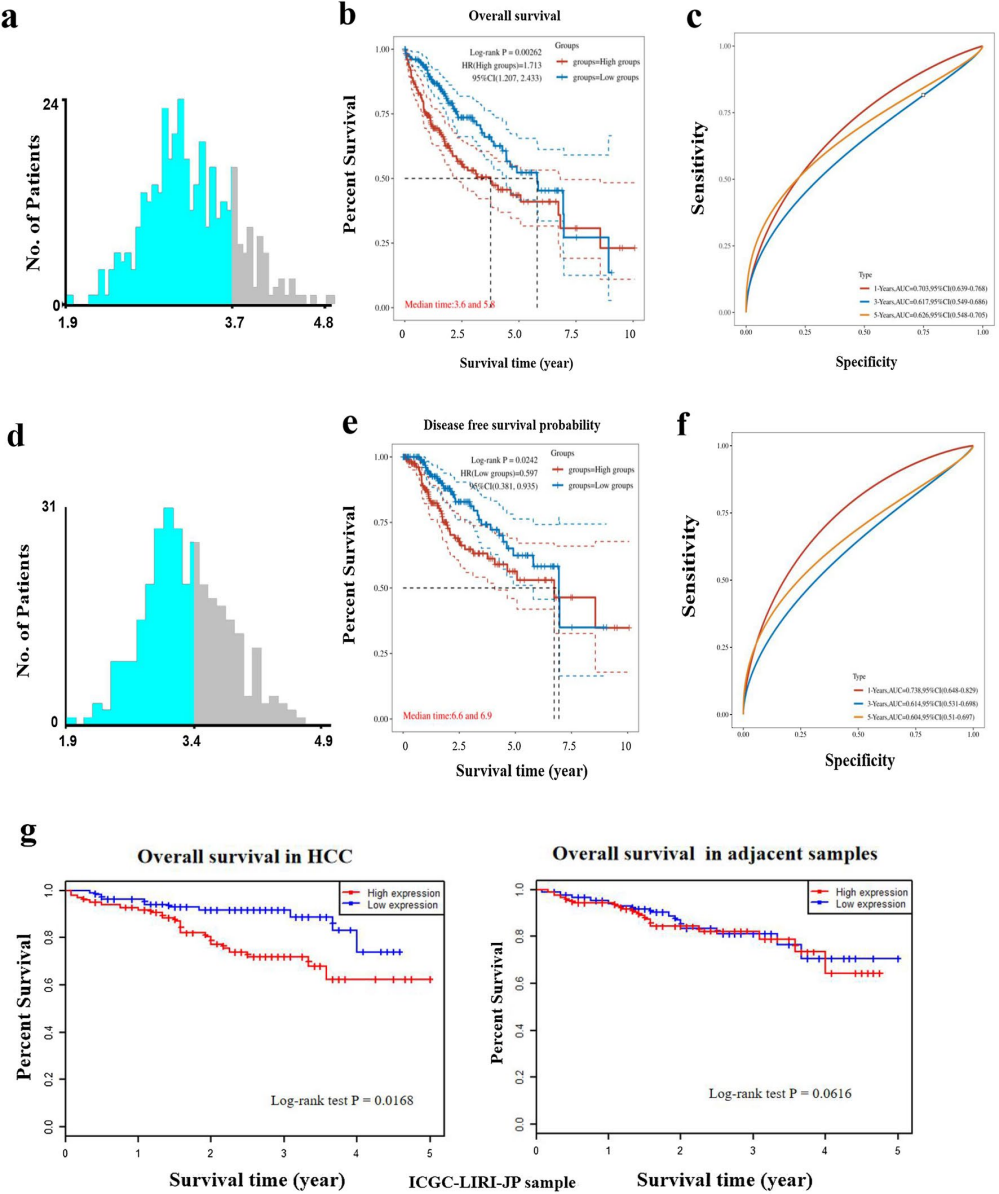

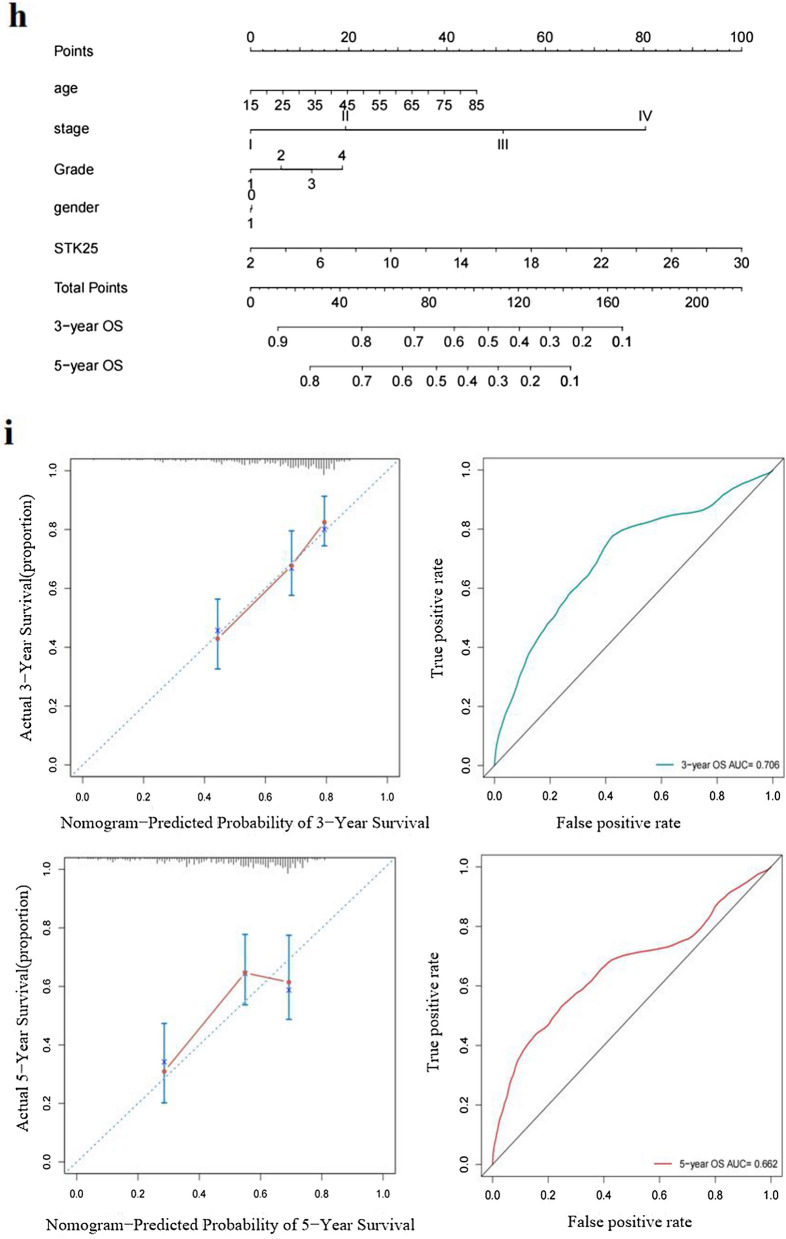
Correlation between STK25 expression and prognosis. **a**, **d** Cutoff value from X-tile according to the overall survival and disease-free survival. **b**, **e** TCGA datasets. Kaplan–Meier survival curves show that high levels of STK25 are associated with poor survival in HCC, not adjacent. **c**, **f** Area under curve to predict survival time. **g** ICGC-LIRI-JP datasets. **h** postoperative prognostic nomogram for patients with HCC. **i** The calibration curve of the nomogram for predicting overall survival (OS) at 3 years and 5 years. Actual OS is plotted on the y-axis; nomogram predicted probability of OS is plotted on the x-axis

As show in Table 2, the expression of STK25 was not associated with patients’ age, Child–Pugh grade, histologic grade and Ishak score (All *p* > 0.05). However, high STK25 expression was significantly correlated with gender, TNM stage, vascular invasion (All *p* < 0.05). Besides, we performed the Cox regression analyses to explore the indicators of OS in HCC. In the univariate model, TNM stage (IV vs I) stage and STK25 expression were significantly related to OS (*p* < 0.05). The following multivariate analyses confirmed that STK25 was independent indicator of unfavorable OS (Table 3). The cutoff values of STK25 were identified by X-tile (Fig. 2a, d). The Kaplan–Meier survival curves with the log-rank test to estimate the relationship between the expression of STK25, overall survival (OS) and disease free survival (DFS) (Fig. 2b, e). More, we can observe the efficacy of STK25 as a prognostic biomarker by AUC (Fig. 2c, f). Obviously, patients with high STK25 expression had a shorter survival time than patients with low STK25 expression (*p* < 0.05). Using an additional database, we further grouped patients with high and low expression of STK25 in cancer tissue for prognostic analysis and obtained consistent results (Fig. 2g), again validating our hypothesis.

**Table 2:** Association between STK25 expression and clinicopathological parameters in patients with HCC in TCGA

Variables	STK25 expression		χ2	*P*
	Low (n = 290) (%)	High (n = 75) (%)		
Gender			5.217	**0.022**
Male	188 (64.8)	59 (78.7)		
Female	102 (35.2)	16 (21.3)		
Age			1.354	0.245
≦65	176 (60.7)	51 (68.0)		
> 65	114 (39.3)	24 (32.0)		
Child–Pugh grade			3.909	0.142
A	179 (61.7)	37 (49.3)		
B–C	17 (5.9)	5 (6.7)		
Unknow	94 (32.4)	33 (44.0)		
TNM stage			10.618	**0.030**
I	139 (47.9)	31 (41.3)		
II	70 (24.1)	14 (18.7)		
III	56 (19.3)	27 (36.0)		
IV	4 (1.4)	0 (0.0)		
Unknow	21 (7.3)	3 (4.0)		
Histologic grade			3.217	0.200
G1–G2	177 (61.0)	53 (70.6)		
G3–G4	108 (37.2)	22 (29.4)		
Unknow	5 (1.8)	0 (0.0)		
Vascular invasion			9.660	**0.008**
No	172 (59.3)	33 (44.0)		
Yes	83 (28.6)	23 (30.7)		
Unknow	35 (12.1)	19 (25.3)		
Ishak score			2.884	0.236
0–4	111 (38.3)	21 (28.0)		
5–6	58 (20.0)	19 (25.3)		
Unknow	121 (41.7)	35 (46.7)		

Statistically significant *p* values are given in bold, *P* < 0.05

**Table 3:** Cox proportional hazards regression model analysis of overall survival

Variables	Univariate analysis		Multivariate analysis	
	HR (95% CI)	*P*	HR (95% CI)	*P*
STK25 (high vs. low)	1.11 (1.06, 1.16)	** < 0.001**	1.10 (1.05, 1.15)	** < 0.001**
Age (> 65 vs. ≦65)	1.01 (0.99, 1.03)	0.181	–	–
Gender (female vs. male)	0.82 (0.56, 1.21)	0.328	–	–
Child–Pugh grade (B–C vs. A)	1.63 (0.73, 3.65)	0.234	–	–
TNM stage(II vs. I)	1.06 (0.52, 2.19)	0.868	0.82 (0.37, 1.83)	0.632
(III vs. I)	1.96 (0.99, 3.88)	0.053	1.92 (0.91, 4.04)	0.087
(IV vs. I)	6.27 (1.86, 21.14)	**0.003**	10.39 (2.85, 37.95)	** < 0.001**
Histologic grade (G3–G4 vs. G1–G2)	1.29 (0.734, 2.26)	0.374	–	–
Invasion(Yes vs. No)	1.67 (0.93, 2.98)	0.083	2.00 (1.04, 3.86)	**0.038**
Ishak score (5–6 vs. 0–4)	1.03 (0.58, 1.83)	0.932	–	–

Statistically significant *p* values are given in bold, *P* < 0.05

Besides, we built a nomogram for predicting the OS of HCC patients in the TCGA cohort. The cancer stage and STK25 are the main factors in the nomogram (Fig. 2h). The calibration curve showed that the reliable in the predicting possible of 3-, 5- years OS in HCC. The black line stand for ideal prediction, and the red line indicates the actual fit. Meanwhile, the 3-, 5- years AUC values were 0.706, 0.662, respectively (Fig. 2i). These results might contribute to efficacy assessment and managing patients.

### STK25 promotes cell proliferation, invasion, and migration in vitro

**Fig. 3:**
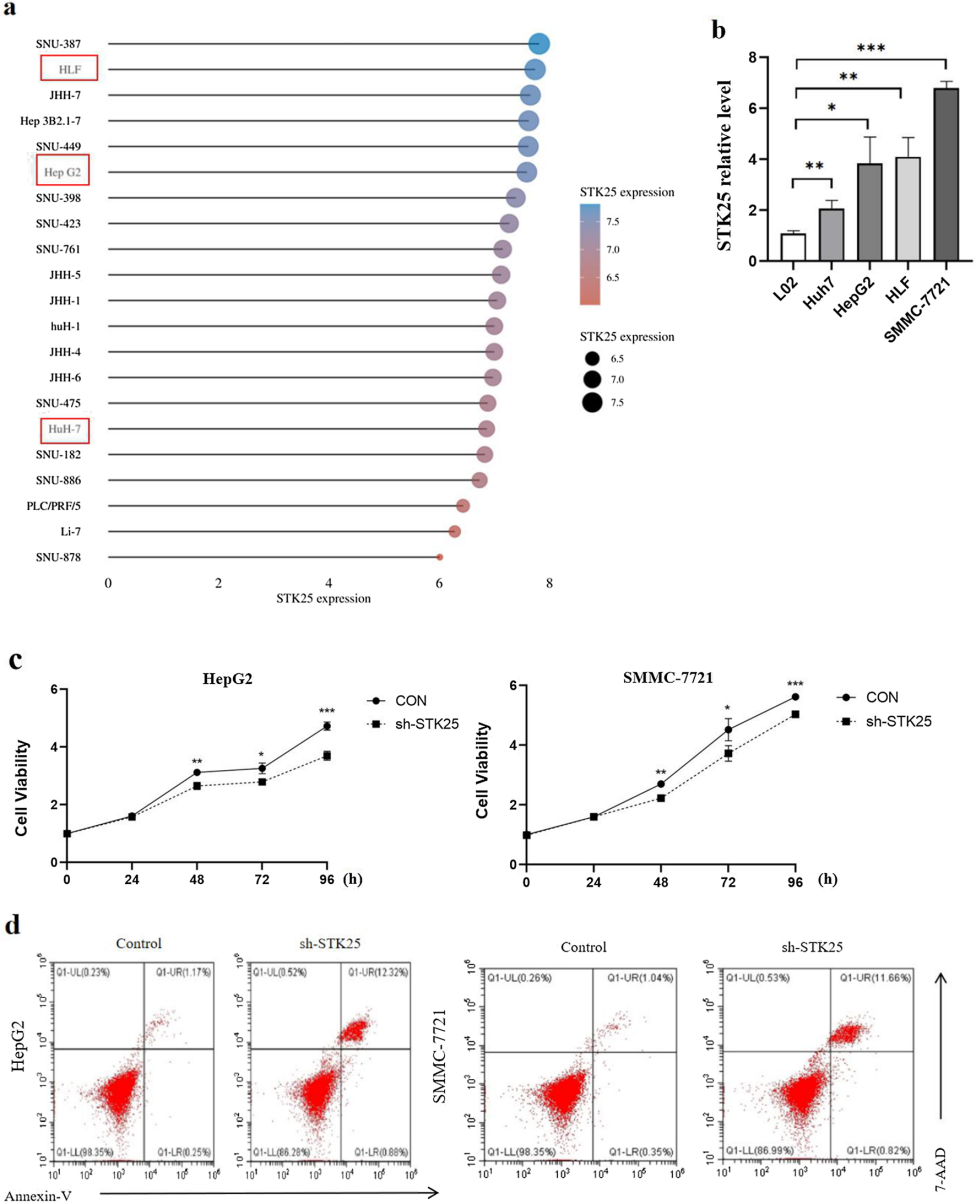

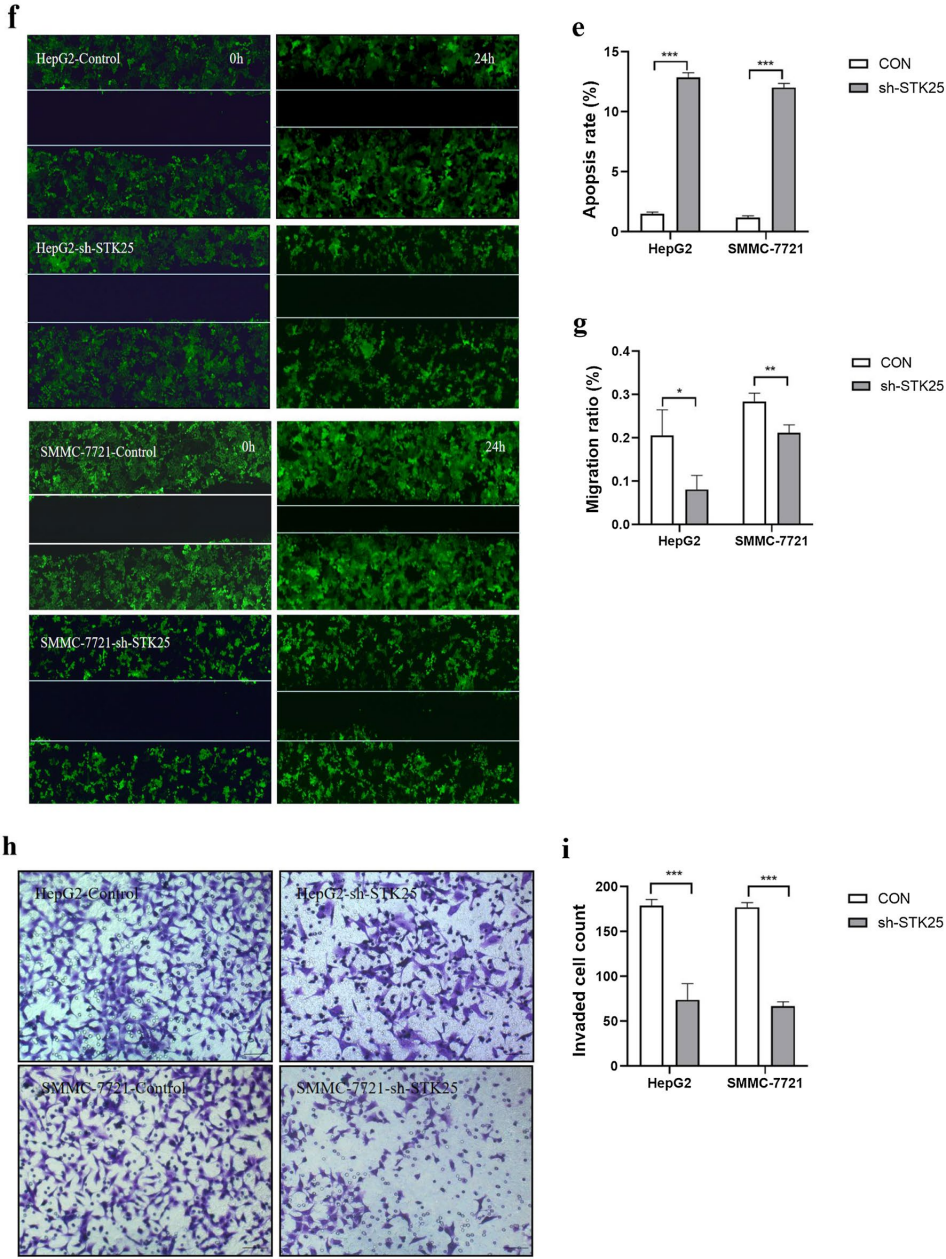
STK25 promotes HCC cell growth. **a** The cell line expression matrix of liver tumors was obtained from the CCLE dataset. The horizontal axis in the figure represents the expression of STK25, the ordinate is for different cell lines, the size of the dot in the figure represents the level of expression, and the different colors also represent the level of expression. **b** Expression of STK25 mRNA in human normal liver HL-7702 (L02) and 4 HCC cell lines (HepG2, Huh7, HLF, SMMC-7721) was measured by qRT-PCR. **c**–**e** Cell viability assay and flow cytometry. depletion of STK25 significantly inhibits cell proliferation and promotes apoptosis. **f**, **g** Wound healing assay. Detection of two time points (0 h, 24 h). The area within the white line is the measured area. Knockdown of STK25 inhibits migration in cell lines. **h** Transwell Invasion assay. Representative images of invasive cells from control and STK25-silenced group. **i** Quantification of cells indicates the invasion ability of STK25-silenced is decreased. CON, control. Data are expressed as mean ± SD. ^*^, *p* < 0.05. ^**^, *p* < 0.01. ^***^, *p* < 0.001. Scale bars, 40 μm

In order to screen suitable HCC cell lines for in vitro experiment, we got the cell line expression matrix from the CCLE dataset (Fig. 3a). Then, the mRNA was determined in the normal liver cell line L02 and the HCC cell lines HepG2, Huh7, HLF and SMMC-7721 (Depends on the growth state of tumor cells). The expression of STK25 was higher in the HepG2, Huh7, HLF and SMMC-7721 cells than in the L02 cells (Fig. 3b), suggesting that cell lines can be used for further study.

To further demonstrate the role of STK25 in HCC, we used shRNA to knockdown STK25. STK25 knockdown cells were seeded into 96-well plates. The cell viability was detected by CCK-8 assay after 0, 24, 48, 72 and 96 h. Compared with the control group, the cell viability of the knockdown group decreased after 48 h, and the difference was statistically significant (*p* < 0.05) (Fig. 3c). Whereas apoptosis increased with STK25 knockdown (*p* < 0.001) (Fig. 3d, e). The result suggests that STK25 plays a positive role in cell activity.

As detected by wound healing after 0 h, 24 h, STK25 knockdown suppressed the migration ability of cells compared with the control group (Fig. 3f, g). The difference in invasive ability after STK25 knockdown was more significant. We observed the invasive ability of the two groups and obtained the same results (Fig. 3h, i).

### STK25 regulates the lipid metabolism pathway in HCC cells

**Fig. 4:**
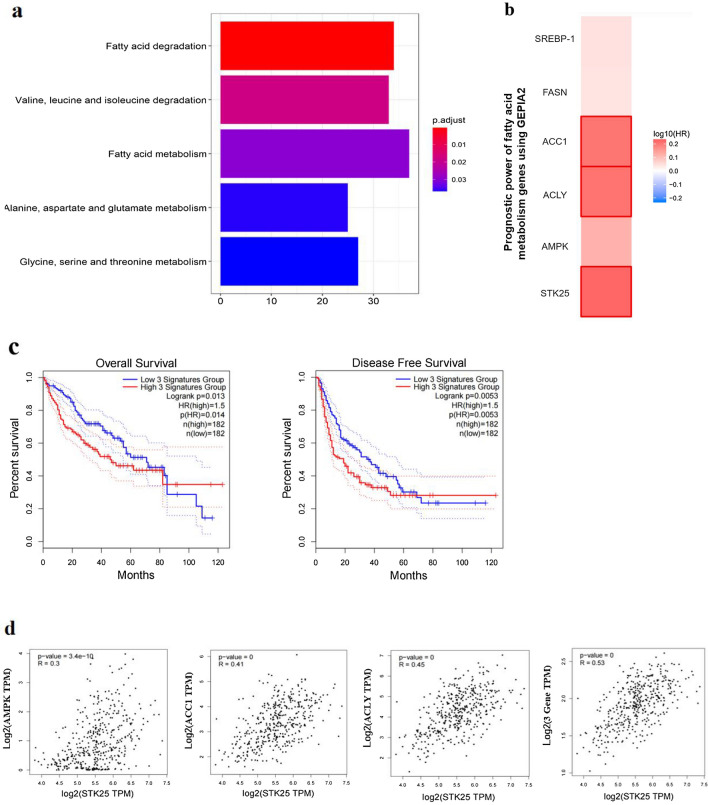

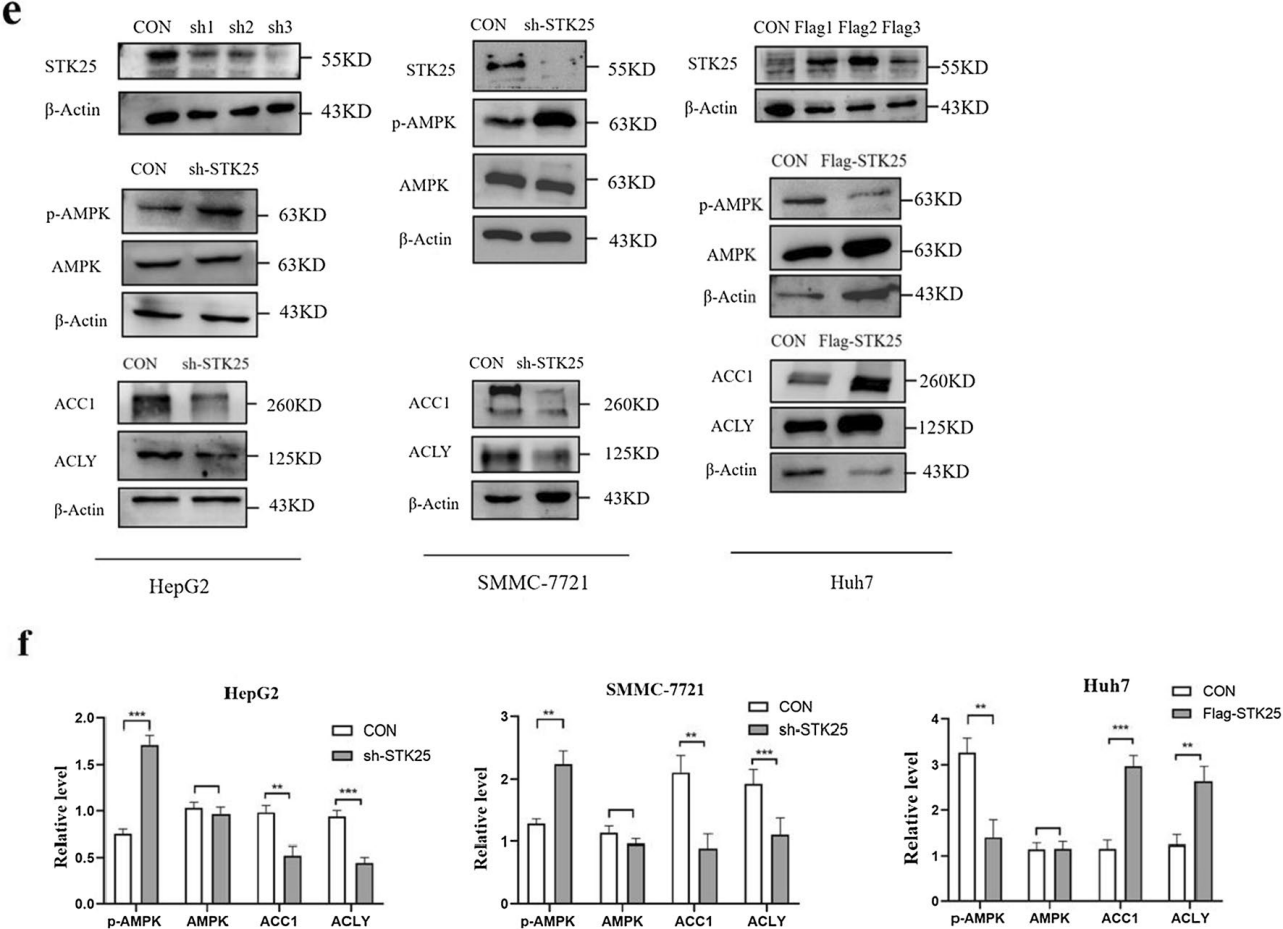
STK25 expression regulates lipid metabolism pathway in HCC cell lines. **a** Significant KEGG pathway terms of DEGs, including fatty acid degradation pathway and fatty acid metabolism pathway. **b**, **c** Kaplan–Meier analysis of overall survival according to the expressions of fatty acid metabolism genes in TCGA data using GEPIA online tool without false discovery rate (FDR) adjustment. The boxes with framed indicated significant results (p < 0.05). **d** Pearson correlation between STK25 and AMPK, ACC1, ACLY. **e** The levels of genes related to lipid metabolism, including p-AMPK, AMPK, ACC1, ACLY were determined in controls, STK25-silenced and STK25-overexpressed HCC cells by western blot. **f** Quantification of western blot for genes related to lipid metabolism. CON, control. sh, knockdown. flag, overexpression. Data are expressed as mean ± SD. ^*^, *p* < 0.05. ^**^, *p* < 0.01. ^***^, *p* < 0.001. Scale bars, 40 μm

There are many differential genes between HCC tissue and paraneoplastic tissue, then we performed functional enrichment analysis on these genes. KEGG signaling pathway enrichment analysis was used to study the differentially expressed genes. According to the screening criteria of *p* < 0.05, five signaling pathways were enriched, as shown in Fig. 4a. They are the fatty acid degradation pathway, the valine, leucine and isoleucine degradation pathway, the fatty acid metabolism pathway, the alanine, aspartic acid and glutamic acid metabolism pathway, aspartate and glutamate metabolism, and glycine, serine and threonine metabolism. Enrichment analyses implicated abnormal lipid metabolism as a potential mechanism in the occurrence and development of HCC. The correlation between key genes of lipid metabolism and prognosis of HCC patients was predicted by GEPIA, we found that ACC1 and ACLY are related to the prognosis of patients (Fig. 4b, c). Although AMPK did not show the correlation with prognosis, ACC1 and ACLY were downstream of AMPK signaling pathway. Analyzing the correlation between STK25 and the other three genes in this pathway, the difference is statistically significant (Fig. 4d).

To determine whether STK25 regulates lipid metabolism in HCC, knockdown of STK25 by three different plasmids (sh1, sh2, sh3) was performed. We selected the sh3 sequence as the most effective. The phosphorylation of AMPK (p-AMPK) protein increased after STK25 knockdown, while the expression of total AMPK remained unchanged. In addition, the expression of downstream ACC1 protein and ACLY protein were decreased compared to that in the control group in HepG2 and SMMC-7721 cell lines, which indicated that lipid synthesis was decreased. The results were opposite after overexpression of STK25 in Huh7 cell line (Fig. 4e, f).

### STK25 interacts with STRN to regulate AMPK pathway

**Fig. 5:**
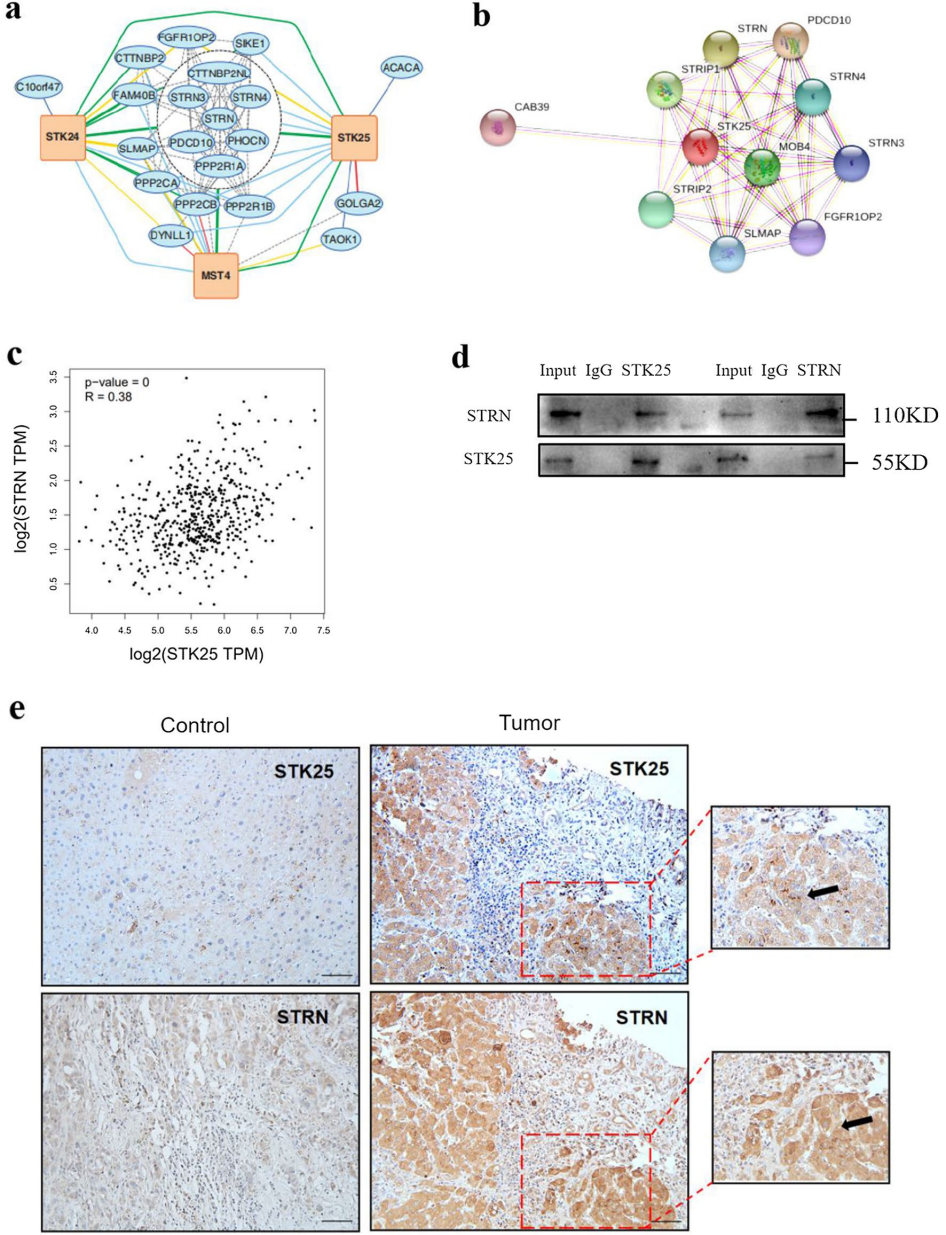

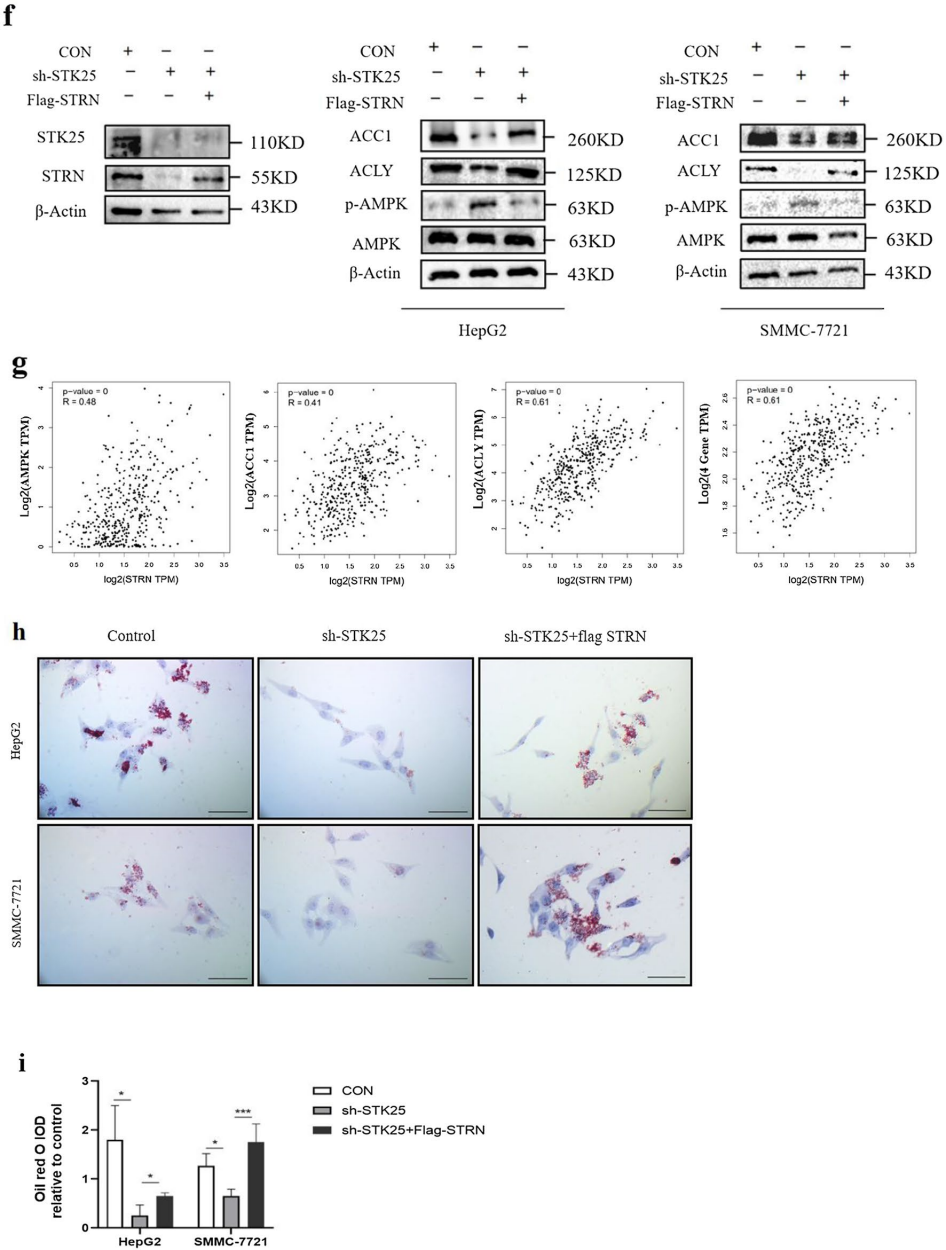
Interaction between STK25 and STRN. **a** Subnetworks of striatum interacting phosphatase and kinase complex (STRIPAK) [17]. **b** Protein networks related to STK25 from String program. **c** Pearson correlation between STK25 and STRN from GEPIA. **d** Determining the interaction between STK25 and STRN by Coimmunoprecipitation. **e** Representative IHC staining images (200×) of STK25 and STRN expression in liver of control and HCC patients, the thick black arrow indicated STK25. **f** STRN was overexpressed in STK25 knockdown cells, and proteins related to AMPK/ACC signaling pathway were detected by western blot. **g** Pearson correlation between STRN and AMPK, ACC1, ACLY. **h**, **i** Oil Red O test. The red color is intracellular lipid droplets. CON, control. sh, knockdown. flag, overexpression. ^*^, *p* < 0.05. ^**^, *p* < 0.01. ^***^, *p* < 0.001. Scale bars, 50 μm

According to previous studies, affinity purification mass spectrometry (AP-MS) was used to obtain the subnetworks of the STK24, STK25 and MST4 kinases, which also constitute the striatum interacting phosphatase and kinase complex (STRIPAK) (Fig. 5a) [17]. The core members of STRIPAK are in the middle circle, and the lines with different colors represent the interactions. We used STK25 as the center, and the protein networks related to STK25 were analyzed with the String program, including STRN (Fig. 5b). Then we used GEPIA to analyze TCGA database and found the correlation, R = 0.38 (Fig. 5c). Further, we confirmed the interaction between them at the protein level by immunocoprecipitation (Fig. 5d). Consistently, IHC staining of consecutive sections of HCC samples also supported these findings. In two consecutive sections of one sample, we observed that a high expression of STK25 was associated with a high expression of STRN in the same location (Fig. 5e).

Intriguingly, we knocked down STK25, transfected Flag-STRN, tested whether STRN overexpression could reverse the changes of AMPK signaling pathway. As shown in Fig. 5f, the rescue experiment partially restored the levels of ACC1 and ACLY. Analyzing the correlation between STRN and the other three genes in this pathway, the difference is statistically significant (Fig. 5g).

Amrutkar et al. found that STK25 is highly expressed in fatty liver, and it promotes the synthesis of fatty acids and the accumulation of lipid droplets in cells [16]. In our study, the change of ACC1 and ACLY are also directly reflected in the accumulation and deposition of lipid droplets in HCC cells. We found that STK25 knockdown limited the accumulation and deposition of lipid droplets, which could be reversed by upregulating STRN (*p* < 0.05) (Fig. 5h, i). Besides, using AMPK inhibitor can also partly reverse the changes of AMPK pathway after STK25 knockdown (Additional file 1: Fig. S1). Therefore, the above results show that STK25 inhibits AMPK signaling pathway by regulating STRN, thereby inducing lipid synthesis in HCC.

### STK25 trigger EMT via the STRN/AMPK/ACC pathway

**Fig. 6:**
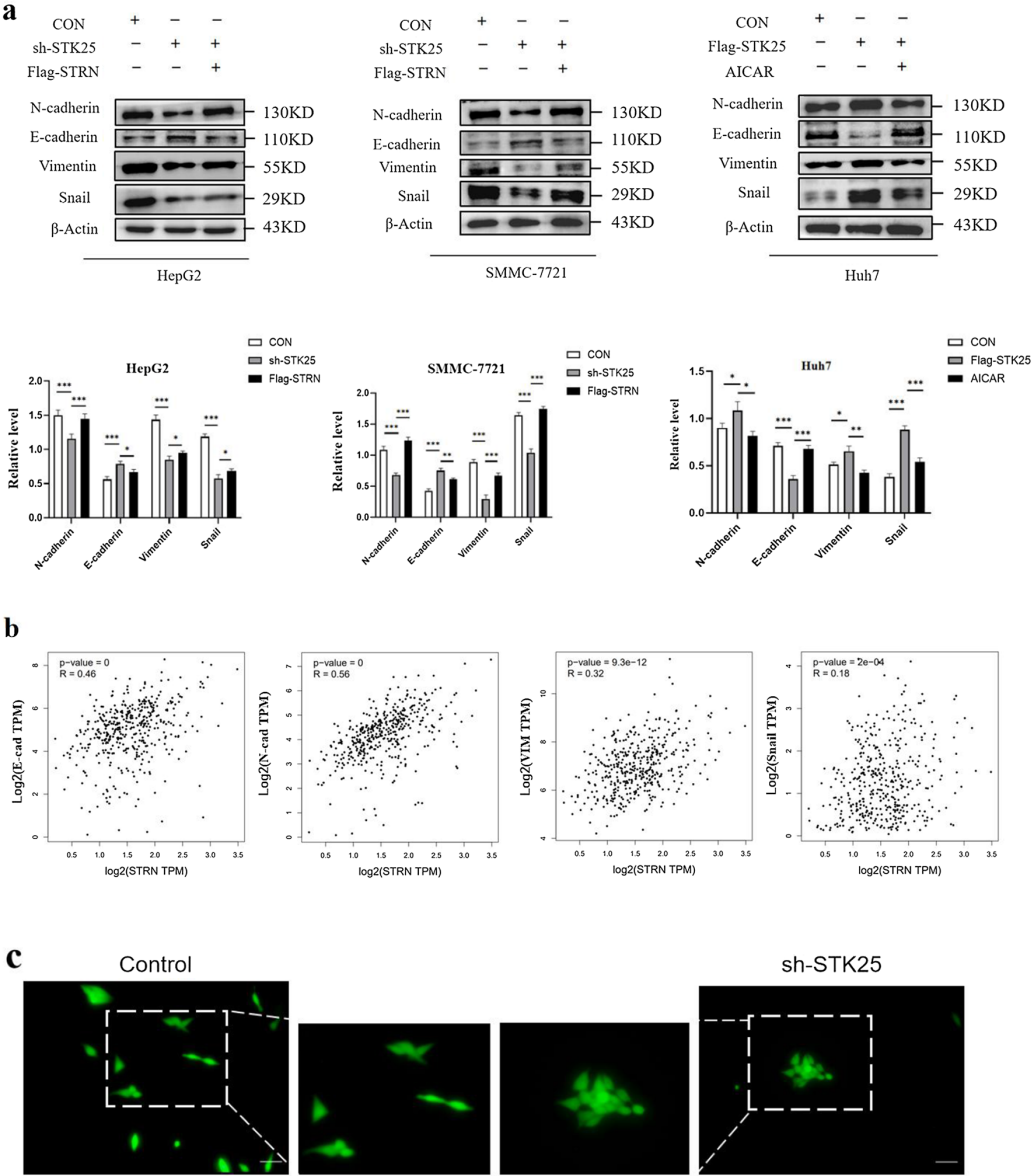

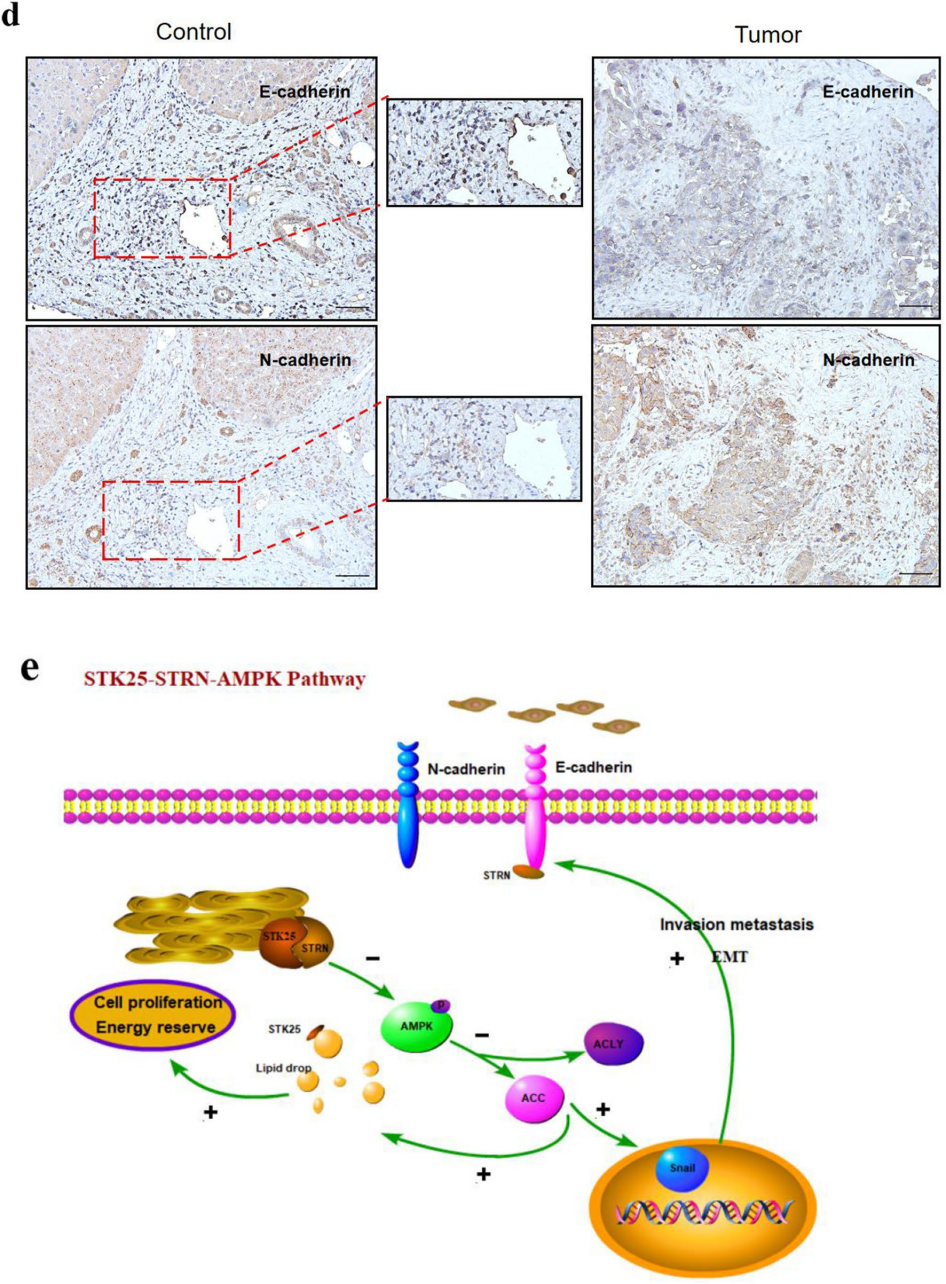
STK25 trigger EMT in HCC cells. **a** Representative western blots and quantification of EMT-related markers in HCC cells. The effect of STK25-silenced was reversed by STRN overexpression in HepG2 and SMMC-7721 cells. The effect of STK25 overexpression was reversed by AMPK agonist in Huh7 cells. **b** Pearson correlation between STRN and EMT-related makers. **c** Cellular morphologic change. The sh-STK25 group showed decreased formation of pseudopodia, leading to an elongated, irregular fibroblastoid morphology, HCC cells gathered into clusters and the dissociation decreased. **d** Representative IHC staining images (200×) of EMT-related markers E-cadherin and N-cadherin in liver of control and HCC patients. **e** Proposed model of STK25 induced biological function in HCC. STK25 inhibits phosphorylation of AMPK by interacting with STRN, thereby up-regulating the expression of ACC1 and ACLY, and stimulating EMT and lipid energy reserve. E-cad, E-cadherin. N-cad, N-cadherin. VIM, vimentin. CON, control. sh, knockdown. flag, overexpression. Data are expressed as mean ± SD. ^*^, *p* < 0.05. ^**^, *p* < 0.01. ^***^, *p* < 0.001

ACC1 levels correlated with metastatic potential in breast involved in changes in induction of EMT [18]. To determine whether STK25 contributes to EMT, we firstly assessed EMT-related protein expression by IHC staining analyses. Deficiency of STK25 led to an epithelial phenotype including an elevated expression of E-cadherin and a downregulated expression of N-cadherin in the HepG2 and SMMC-7721 cell lines, which could be reversed by STRN overexpression (Fig. 6a). In contrast, we used AMPK agonist (AICAR) to change the effect of STK25 overexpression in Huh7 cell line, which echoed the previous results. Then TCGA database was used to support the correlations between STK25 and EMT markers, and the correlations were consistent with the previous results (Fig. 6b). During the process, the sh-STK25 group showed decreased formation of pseudopodia and cell adhesion (Fig. 6c). Consistently, IHC staining of consecutive sections of HCC samples also supported these findings (Fig. 6d). In Fig. 6e, we showed the mechanism diagram of the experiment, STK25/STRN/AMPK/ACC1 pathway.

### Inhibition of STK25 suppresses tumor growth and lipid synthesis in vivo

**Fig. 7:**
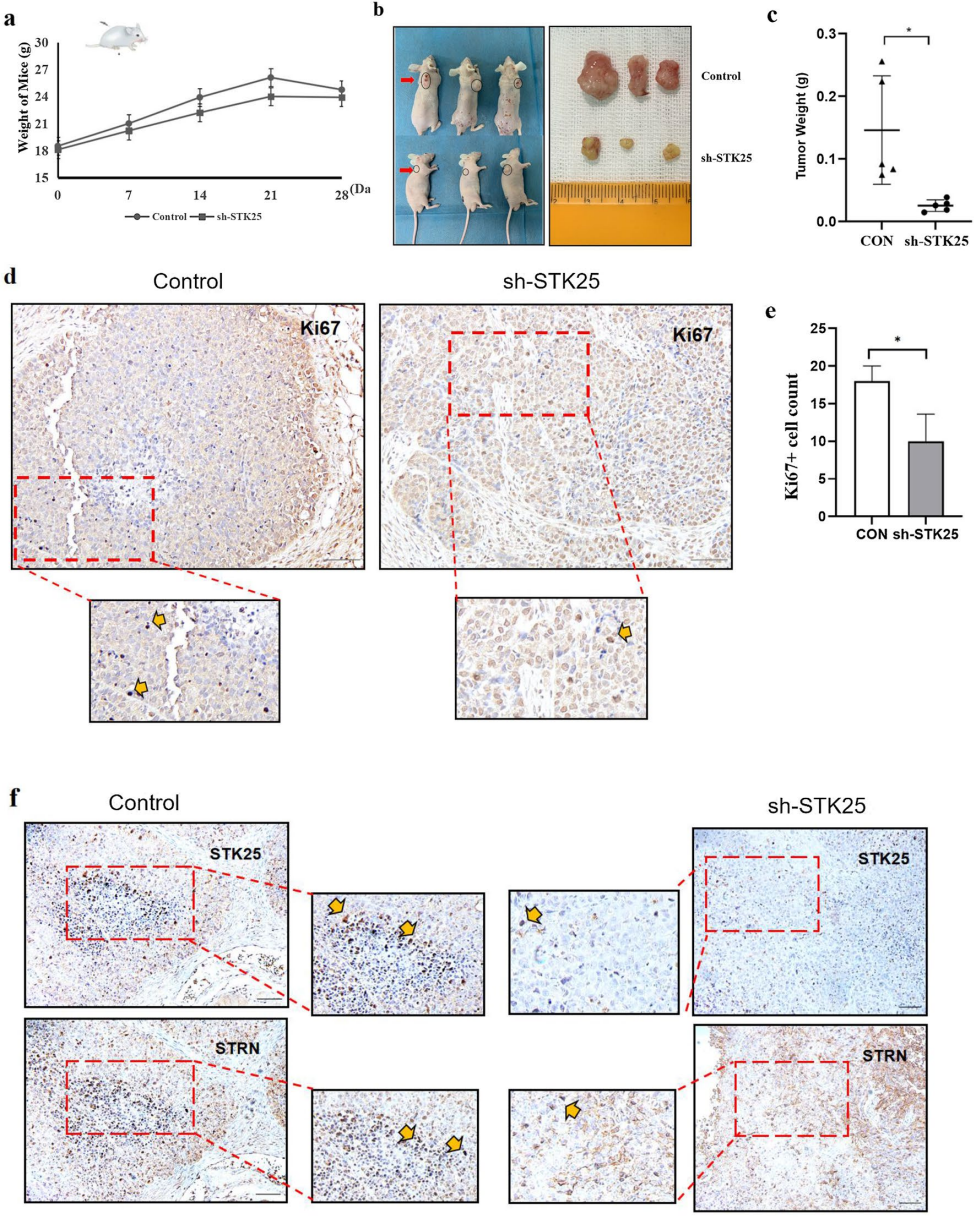

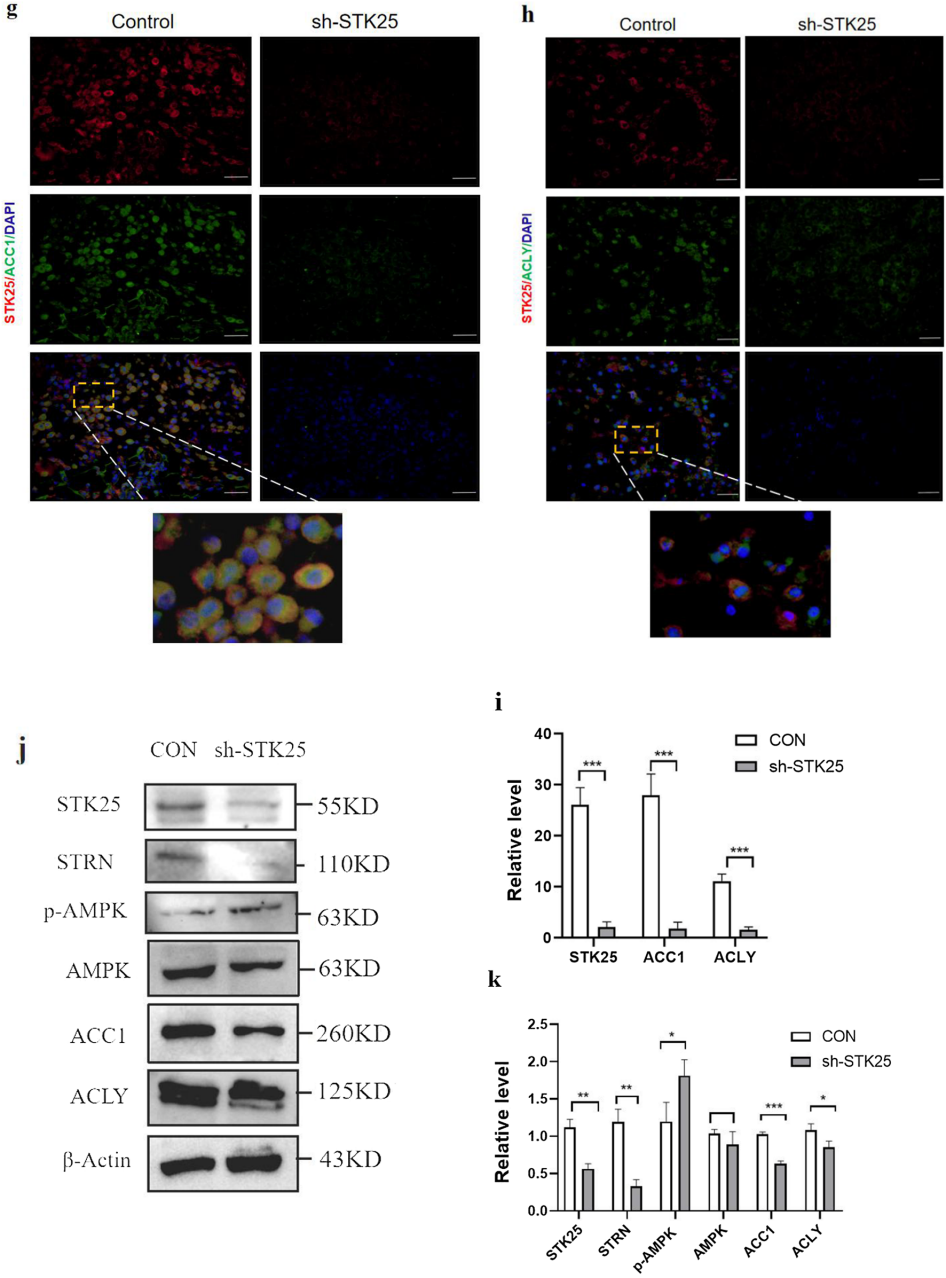

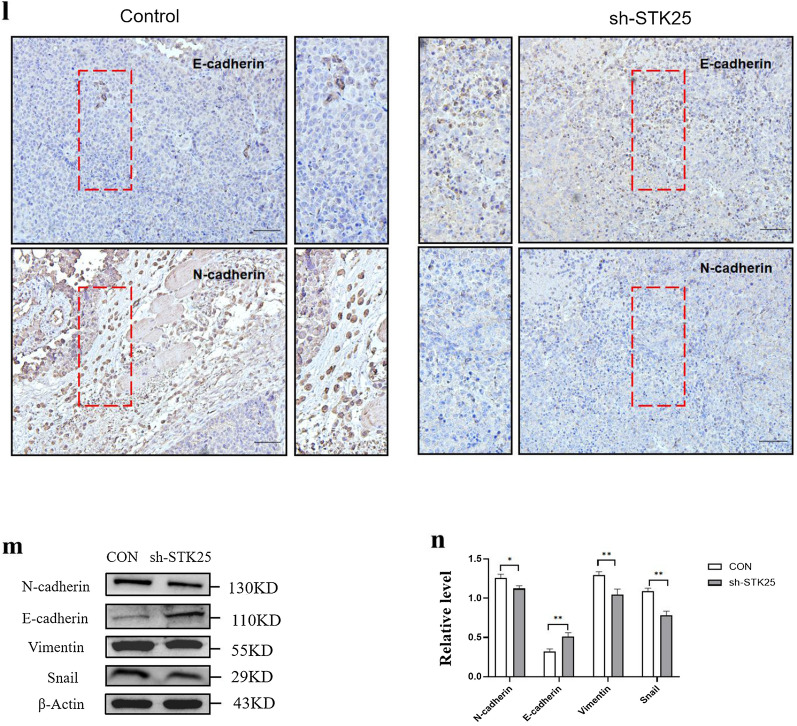
STK25 knockdown inhibits tumor growth and lipid metabolism pathway in a xenograft mouse model. **a** Weight of mice. **b** Representative images of tumor-bearing mice implanted with HepG2-shControl or HepG2-shSTK25 cells (Left). Tumor masses were harvested from the corresponding xenografts on Day 28 (Right). **c** Tumor weights. **d** Representative images of Subcutaneous tumor from control and sh-STK25 groups immunostained with Ki67 (200×), the thick yellow arrow indicated Ki67. **e** Quantification of Ki67 + cells. (**f**) Representative IHC staining images (200×) of STK25 and STRN expression. **g**, **h** Representative images of subcutaneous tumor from control and sh-STK25 groups immunostained with STK25 (red), ACC1/ACLY (green) and DAPI (blue). The depletion of STK25 was accompanied by the decrease of ACC1 and ACLY. (**i**) Quantification of immunofluorescence for STK25, ACC1, ACLY. **j** Representative western blots of STK25 expression and genes related to lipid metabolism. **k** Quantification of western blot for STK25 and genes related to lipid metabolism fold change in sh-STK25 compared with that in control group. **l** Representative IHC staining images (200×)of EMT-related markers E-cadherin and N-cadherin in subcutaneous tumor. **m**, **n** Representative western blots and quantification of EMT-related markers in subcutaneous tumor. CON, control. Data are expressed as mean ± SD. ^*^, *p* < 0.05. ^**^, *p* < 0.01. ^***^, *p* < 0.001. Scale bars, 50 μm

To further investigate the correlation between STK25 mediated regulation of lipid metabolism and tumorigenesis, human HCC xenografts in nude mice were established by subcutaneous injection of HepG2-shSTK25 or Control cells. Compared with the control group, sh-STK25 group had no effect on the weight of the mice (Fig. 7a). But tumor growth and weight in the sh-STK25 group decreased significantly compared with the Control group (*p* < 0.05) (Fig. 7b, c).

To confirm the proliferation ability of tumor cells in vitro, we detected the expression of Ki67 and found there were less Ki67 + cells in sh-STK25 group (Fig. 7d, e). Additionally, in two consecutive sections of one sample, we also observed that a high expression of STK25 was associated with a high expression of STRN in the same location (Fig. 7f).

In order to more intuitively reflect the changes of lipid metabolism pathway and EMT after STK25 knockdown, the tumor tissues were also analyzed to evaluate the differences in STK25 and lipid metabolism. Immunofluorescence of subcutaneous tumor was performed to compare ACC1 and ACLY expression. The expression was significantly decreased by STK25 depletion (Fig. 7g–i). By western blot, lower STK25 expression in sh-STK25 group was associated with down-regulation of p-AMPK, ACC1, ACLY compared with sh-Control group (Fig. 7j, k). Moreover, the results of EMT in vivo were consistent with those in vitro (Fig. 7l–n). These observations indicate that the silencing of STK25 inhibits tumor growth by decreasing lipid synthesis in HCC.

## Discussion

In recent years, nutrition and metabolic therapy for cancer has received widespread attention. Nutritional intervention affects the metabolic process of tumors, mainly by regulating the intake of carbohydrates, protein/amino acids and fat; this regulation involves reducing the supply of glucose, increasing the supply of protein, and choosing appropriate fatty acids and ketogenic diet to ultimately inhibit the growth of tumor cells and even induce their death [19, 20].

Due to the aberrations of tumor cells, the energy required for proliferation and invasion may be different from that of normal cells, such as the Warburg effect. The Warburg effect, also known as aerobic glycolysis, is a critical feature of cancer cells, which means that cancer cells have the tendency to employ glycolysis, even in the presence of sufficient oxygen [21, 22]. Blocking or reducing this specific process can improve the effects of antitumor therapy. Lipid metabolism is also involved in many cellular processes (e.g. cell survival and apoptosis), and its disruption is closely related to the malignant growth of cancer [23]. In rapidly proliferating cells, citrate is cleaved by ACLY to produce cytosolic acetyl coenzyme A, which is the beginning of lipid synthesis. ACC plays the role of rate-limiting enzyme in lipid synthesis. ACLY and ACC are always upregulated to meet the demand for membrane expansion in cancer [24, 25]. Studies have shown that activation of AMPK-ACC pathway can decrease lipid metabolism and thus induce pancreatic cell apoptosis [24]. The liver is an important organ of lipid metabolism, so whether there is a unique lipid metabolism mode in liver cancer cells deserves further study.

As an important signaling molecule, STK25 has been widely studied in nonalcoholic steatohepatitis/nonalcoholic fatty liver disease (NASH/NAFLD). Previous studies proved the increased expression of STK25 in NASH mouse and human liver. It has been confirmed that NASH mice have pathological changes such as hepatocyte injury, steatosis and fibrosis, but these pathological changes are not significant in STK25 knockout mice, and the severity of liver injury detected by liver puncture in NASH patients is consistent with the STK25 mRNA level [6]. In addition, upregulated STK25 inhibited β-oxidation and triglyceride efflux by inhibiting the lipolysis process, leading to the deposition of lipid droplets in the liver. The above effects were reversed in the STK25 knockout group [16, 26]. Targeted transport of STK25 antisense oligonucleotide (STK25 ASO), which could selectively bind to the Asialoglycoprotein receptor (ASGPR) expressed on the surface of hepatocytes, reduced oxidative stress in the hepatocytes, improved mitochondrial function and inhibited the expression of adipogenic genes and Acetyl-CoA carboxylase (ACC) in the liver, thus delaying the development of NAFLD [27]. It is obvious that the above changes also exist in HCC.

In a recent study, Yeshwant Kurhe [28] found the depletion of STK25 could suppressed liver tumor growth and hinders the development of NASH-related HCC, through STAT3, ERK1/2, and p38 signaling pathway. In our study, we revealed a close correlation between STK25 and the stage and prognosis of patients with HCC through the TCGA and GEO databases. Furthermore, we verified an abnormal increase in STK25 in the tissues of patients with HCC. To further confirm the interaction between STK25 and lipid metabolism, we knocked down STK25 and found that the AMPK signaling pathway was activated, and lipid synthesis was decreased, accompanied by changes in the biological function of HCC cells. Furthermore, we found that upregulation of STRN reversed the effects of STK25 knockdown, which suggested that STK25 inhibited the phosphorylation of AMPK by activating STRN, then up-regulates ACC1 and finally triggers EMT. The results of the two studies are consistent, but we explored the potential mechanism of NASH-related HCC from two different signaling pathways.

Currently, chemotherapy still represents a primary HCC treatment strategy, but there are low response rate and high therapeutic resistance, resulting in shorter survival of patients with HCC. Therefore, new treatment strategies should be based on the two aspects. It has been shown that non-coding RNAs (ncRNAs) could affect the response to chemotherapy in HCC by modulating proliferation, apoptosis, tumor cell migration, and autophagy [29, 30]. Actually, non-coding RNAs (ncRNAs) can target multiple signaling pathways, so we reasonably speculate that STK25 may also be regulated by ncRNAs and decrease the sensitivity of chemotherapy. In addition, there are many natural compounds in the market. Phytochemicals (such as coumarin) and marine natural products have been evaluated the potential protective mechanisms against cancer. The mechanisms include promoting cell apoptosis and inhibition of cell metabolism [31–33], which are very similar to the effect of STK25. If we could find their correlation with STK25, we believe that the research will be more in-depth and innovative.

## Conclusions

In conclusion, our results demonstrated that STK25 interacted with STRN to regulates the energy reserve and EMT via lipid metabolism reprogramming. Accordingly, high expression of STK25 may be associated with HCC patients and poor prognosis, which implicates STK25 could be a potential target for lipid metabolism in cancer therapy.

## Supplementary Information

**Additional file 1: Fig. S1** The effect of STK25-silenced was reversed using AMPK inhibitor (Compound C) and detected by western blot. Com.C, compound C.

## Data Availability

The datasets used and/or analyzed during the current study are available from the corresponding author on reasonable request.

## Abbreviations

STK25: Serine/threonine protein kinase 25
STRN: Striatin
NAFLD: Nonalcoholic fatty liver disease
NASH: Nonalcoholic steatohepatitis
HCC: Hepatocellular carcinoma
GCKIII: Germinal center kinase subfamily III
KEGG: Kyoto Encyclopedia of Genes and Genomes
DEGs: Differentially expressed genes
GO: Gene Ontology
ASGPR: Asialoglycoprotein receptor
ACLY: ATP citrate lyase
ACC: Acetyl-CoA carboxylase
E-cad: E-cadherin
N-cad: N-cadherin
VIM: Vimentin
OS: Overall survival
DFS: Disease free survival
ncRNAs: Non-coding RNAs
