# Supplementary figures and images for: STK25 enhances hepatocellular carcinoma progression through the STRN/AMPK/ACC1 pathway
Source: Cancer Cell Int. 2022 Jan 5;22:4. doi: 10.1186/s12935-021-02421-w (PMC8734210; doi:10.1186/s12935-021-02421-w)

**Fig. S1**

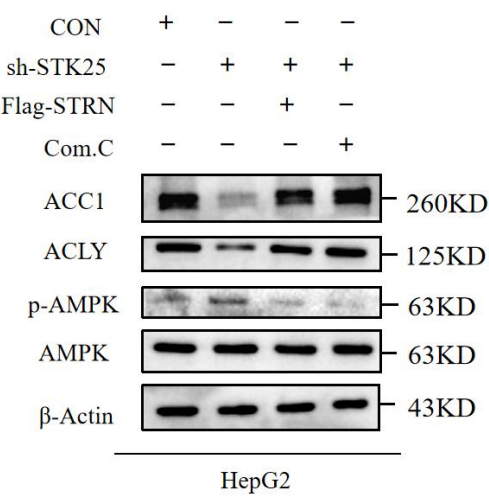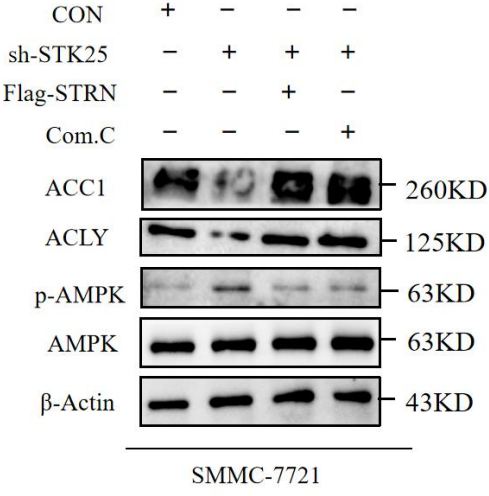

Supplement: Supplementary file 1 — Additional file 1: Fig. S1 The effect of STK25-silenced was reversed using AMPK inhibitor (Compound C) and detected by western blot. Com.C, compound C. [file 12935_2021_2421_MOESM1_ESM.pdf]
